# Interactions between muscle stem cells, mesenchymal-derived cells and immune cells in muscle homeostasis, regeneration and disease

**DOI:** 10.1038/cddis.2015.198

**Published:** 2015-07-23

**Authors:** J Farup, L Madaro, P L Puri, U R Mikkelsen

**Affiliations:** 1 Section for Sports Science, Institute of Public Health, Aarhus University, Aarhus, Denmark; 2 Sanford-Burnham Medical Research Institute, Sanford Children's Health Research Center, La Jolla, CA, USA; 3 IRCCS Fondazione Santa Lucia, Rome, Italy; 4 Institute of Sports Medicine, Department of Orthopaedic Surgery M, Bispebjerg Hospital and Center for Healthy Aging, Faculty of Health and Medical Sciences, University of Copenhagen, Copenhagen, Denmark

## Abstract

Recent evidence has revealed the importance of reciprocal functional interactions between different types of mononuclear cells in coordinating the repair of injured muscles. In particular, signals released from the inflammatory infiltrate and from mesenchymal interstitial cells (also known as fibro-adipogenic progenitors (FAPs)) appear to instruct muscle stem cells (satellite cells) to break quiescence, proliferate and differentiate. Interestingly, conditions that compromise the functional integrity of this network can bias muscle repair toward pathological outcomes that are typically observed in chronic muscular disorders, that is, fibrotic and fatty muscle degeneration as well as myofiber atrophy. In this review, we will summarize the current knowledge on the regulation of this network in physiological and pathological conditions, and anticipate the potential contribution of its cellular components to relatively unexplored conditions, such as aging and physical exercise.

## Facts

- In skeletal muscle regenerative disorders (e.g., muscular dystrophies) as well as age (sarcopenia)- or disease (cachexia)-related decline in muscle mass and function, there is an impairment of the regenerative potential, which correlates with a progressive replacement of contractile mass with fibrotic and adipose tissue.
- Mesenchymal-derived cells, such as Sca1^+^/PDGFR*α* ^+^ fibro-adipogenic progenitors (FAPs), reside in the interstitial space in skeletal muscle and can contribute either to muscle regeneration or to fibrosis and fat deposition.
- Functional interactions between muscle stem cells (satellite cells), FAPs and cells from the inflammatory infiltrate have recently been reported and appear to determine the ability of skeletal muscle to regenerate or undergo fibro-adipogenic degeneration.
- Ectopic adipose tissue in skeletal muscle is associated with impaired insulin sensitivity and muscle function.

## Open Questions

- Are the interactions between satellite cells, FAPs and inflammatory cells relevant in the pathogenesis of neuromuscular diseases?
- Are the interactions between satellite cells, FAPs and inflammatory cells implicated in the functional decline of muscles during cachexia and sarcopenia?
- Are the interactions between satellite cells, FAPs and inflammatory cells implicated in the control of muscle growth and homeostasis during exercise?
- Does an increased ectopic fat deposition in skeletal muscle, from adipogenic differentiation of FAPs, alter the systemic metabolic profile?
- Could the functional network between satellite cells, FAPs and inflammatory cells provide potential targets for pharmacological interventions toward promoting compensatory regeneration in muscular disease, countering age-related or cachexia-mediated muscle atrophy, or improving the response to exercise and the metabolic profile?

Reduction of muscle mass is typically observed at late stages of many neuromuscular diseases, during aging, inactivity and chronic systemic disorders (i.e., diabetes, cancer, rheumatoid arthritis (RA) and chronic obstructive pulmonary disease (COPD)) and is closely associated with impairment of metabolic control collectively worsening the recovery of the primary disease.^1, 2, 3, 4^

The endogenous regenerative potential of skeletal muscle provides a compensatory response against muscle loss; however, this response cannot support continuous muscle regeneration in chronic conditions. The regenerative capacity of skeletal muscle relies on muscle stem cells (satellite cells (SCs)), which proliferate in response to exercise to facilitate muscle growth and remodeling, or following myotrauma to repair the injured muscle.^5, 6, 7, 8^ Recent works identified mesenchymal progenitor cells termed fibro-adipogenic progenitors (FAPs) that provide functional support to SCs; however, these cells might also turn into a source of ectopic fat deposition and fibrosis in skeletal muscle.^9, 10, 11, 12, 13, 14, 15^ Although their role in humans is not fully understood, a cellular population phenotypically and functionally similar to mouse FAPs has been isolated from human muscle.^14^ FAP activity is regulated by physical and functional interactions with myofibers and SCs^10^ as well as cytokines released from innate immune cells.^11^ Moreover, key regulatory intracellular networks that control FAP lineage and function in regenerating muscles of normal and dystrophic mice have recently been reported.^16^

This review summarizes the current knowledge on the role played by the cellular network composed by SCs, FAPs and the inflammatory infiltrate (e.g., macrophages and eosinophils) during physiological and pathological perturbations of muscle homeostasis.

## Muscle Stem Cell, SC and FAP niche, Interaction with Immune Cells and Contribution to Intramuscular Adipose Tissue

Maintenance of muscle mass depends on the integrity of the regenerative machinery, which is composed of SCs and other mononucleated cell types,^17, 18, 19, 20, 21, 22, 23^ although, the direct contribution of SCs to myofiber hypertrophy and maintenance remains controversial.^19, 24, 25, 26^

**Figure 1:**
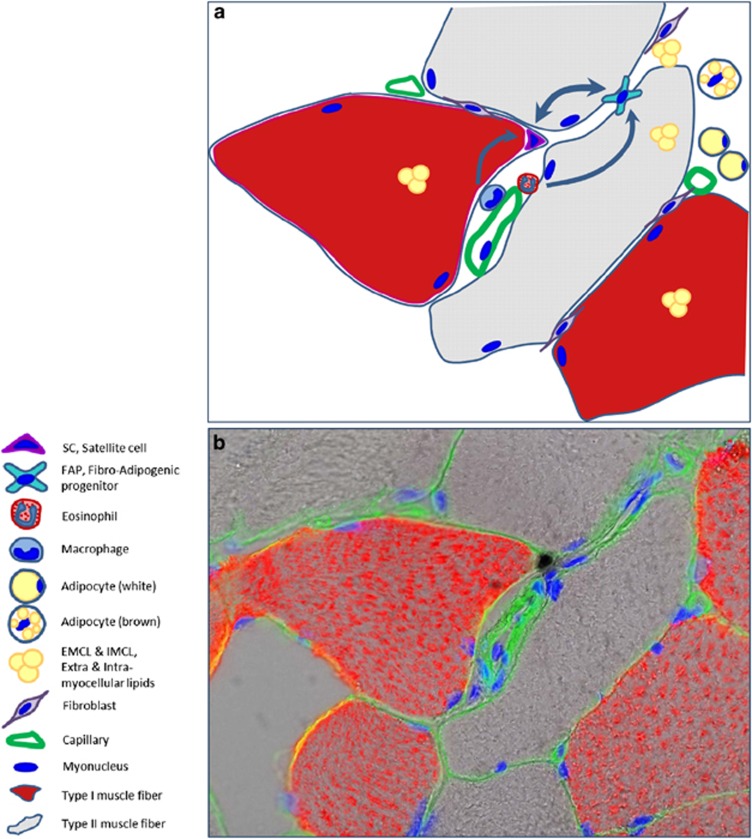
(**a**) Schematic illustration of localization of satellite cell, FAP, macrophage and eosinophil in relation to muscle fibers and capillaries. Compare with image in **b**. Sizes of individual cells are not drawn to scale. (**b**) Immunohistochemical staining of human muscle biopsy cross-section with antibodies against Pax7 (brown), laminin (green) and MHCI (red). Nuclei are stained blue with DAPI

**Figure 2:**
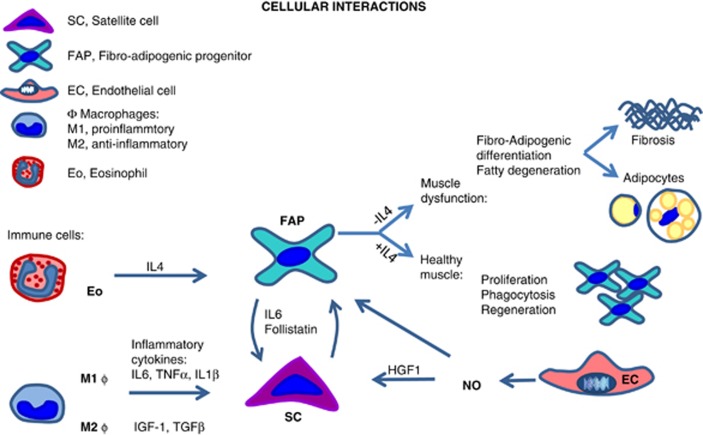
Schematic illustration showing cellular interactions in skeletal muscle between immune cells, SCs, FAPs and endothelial cells with indication of some known mediators of these interactions (based on results from Heredia *et al.*,^11^ Mozzetta *et al.*,^12^ Tardif *et al.*,^46^ Moyer and Wagner^60^ and Snijders *et al.*^182^). For further details see text. Abbreviations: HGF, hepatocyte growth factor; IL, interleukin; IGF-1, insulin-like growth factor 1; NO, nitric oxide; TNF*α*, tumor necrosis factor *α*; TGF*β*, transforming growth factor *β*

SCs are located beneath the basal lamina – the anatomical niche – and their activity is regulated by interactions with cellular components of the 'functional niche' – FAPs, immune as well as vessel-derived cells (Figures 1 and 2).

**Figure 3:**
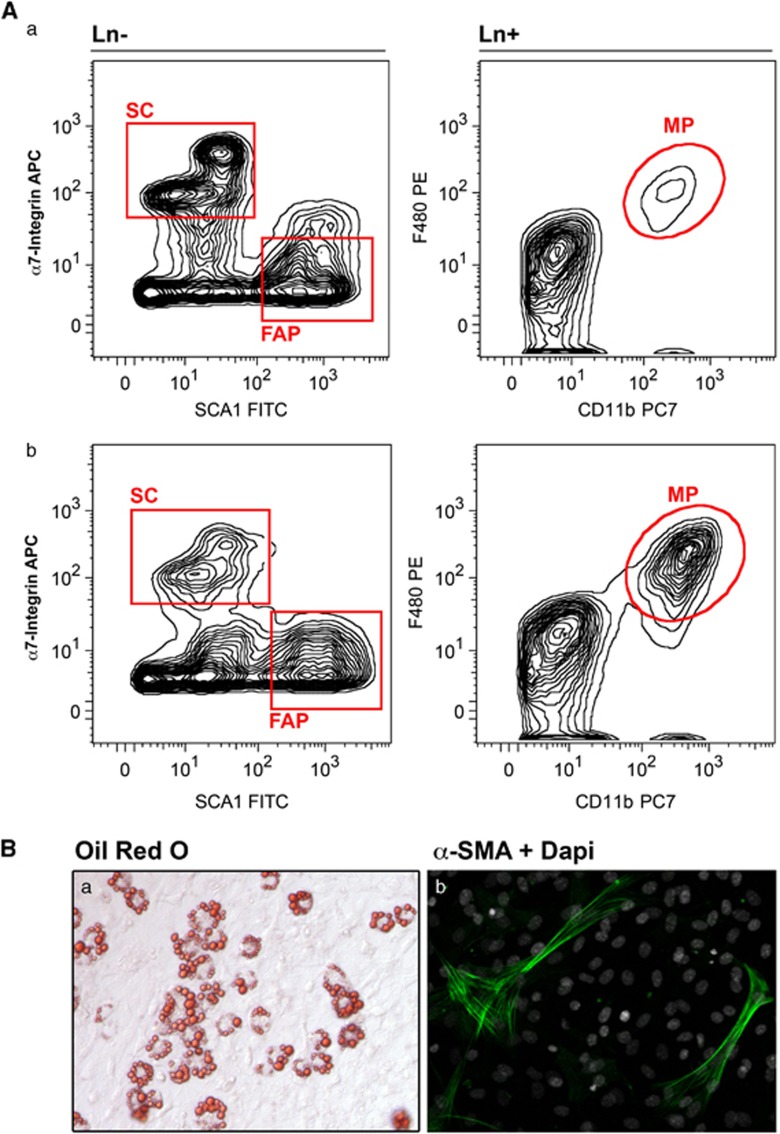
Mononuclear cell isolation procedure from skeletal muscle composed of mechanical and enzymatic digestion, filtration, blocking, antigen labeling and finally multiple parameter FACS to sort out selected cell populations. (**A**) Representative plots showing FACS strategy to sort lineage-negative (Ter119^−^ CD45^−^ CD31^−^) SCs (*α*7 integrin^+^) and FAPs (Sca1^+^) as well as lineage-positive macrophages (MPs, CD11b^+^ F480^+^) from skeletal muscle of healthy (a) and mdx (b) mice. (**B**) Representative images of adipogenic (Oil Red O (a)) and fibrogenic (*α*-smooth muscle actin (b)) phenotype of FAPs during differentiation

FAPs are non-myogenic, interstitial, mesenchymal progenitors that can be isolated by virtue of the absence of SC surface markers and by the expression of platelet-derived growth factor receptor alpha (PDGFR*α*)^+^^9^ or stem cell antigen 1 (Sca1)^+^^10^ (Figure 3). When isolated from regenerating muscles FAPs exhibit the remarkable property of promoting SC cell proliferation and differentiation in co-culture.^9, 10^ However, FAPs also possess an intrinsic adipogenic and fibrogenic potential manifested in culture by exposure to adipogenic conditions,^9, 10^ indicating a potential contribution of FAPs to fibrotic and adipose accumulation in diseased muscles (Figure 2). In addition to formation of ectopic fat and fibrous tissue, abnormal bone formation also occurs under some circumstances in muscle, termed heterotopic ossification,^27^ raising the possibility that the term FAPs does not fully cover their potential roles.^28^

Two recent studies have identified both the anatomical niche and paracrine cues from innate immune cells as signals that regulate FAP lineage and activity.^9, 11^ For instance, IL-4 released by eosinophils has been shown to be a key mediator of FAP fate (Figure 2).^11, 15^ Eosinophils provide an abundant source of IL-4/IL-13 in different conditions.^11, 29, 30, 31^ Chemotaxis of eosinophils to skeletal muscle and release of IL-4/IL-13 is observed in different conditions, such as muscle injury^11^ or exercise,^29^ suggesting that substantial (i.e., chemically induced muscle damage) or subtle (i.e., skeletal muscle exercise) perturbations of skeletal muscle homeostasis may both stimulate FAP-mediated activation of SCs. Inactivation of IL-4-mediated signaling or eosinophil chemotaxis stimulates the adipogenic differentiation of FAPs.^11, 15^ Likewise, interactions between FAPs and myofibers or SCs also regulate the FAP-mediated adipogenesis.^9, 10^ Finally, FAP activity is regulated by growth factors (e.g., insulin-like growth factor 1 (IGF-1), hepatocyte growth factor (HGF)), follistatin and nitric oxide (NO), which are secreted from FAPs themselves, endothelial cells and M1/M2 macrophages^11, 32, 33, 34, 35, 36, 37, 38^ (Figure 2).

In turn, FAPs provide a source of cytokines that regulate SC activity. For instance, FAP-derived IL-6 (LM and PLP unpublished data) activates the signal transducer and activator of transcription 3 (STAT3) in SC.^10, 39, 40, 41^ This pathway is involved in SC activation and its dysregulation appears implicated in age-dependent reduced regenerative capacity.^42^ FAPs also provide a substantial source of follistatin secretion, with a tenfold higher follistatin expression in FAPs compared to SCs.^12^ Follistatin is the physiological antagonist of the negative regulator of muscle mass, myostatin^12, 43, 44^ and besides preventing myofiber atrophy, it may influence myofiber regenerative capacity through directly targeting the SCs. Interestingly, in aged humans the reduced SC proliferation following exercise is associated with increased co-localization of myostatin in SCs of aged muscle.^21^ Thus, the FAP-mediated release of follistatin may play a role in SC regulation in rodent as well as in human muscle.

These results suggest that FAPs from regenerating muscles retain a functional bipotency, whose resolution is dependent on anatomical factors (e.g., interactions with myofibers, other cell types or extracellular matrix), systemic factors and local concentrations of soluble cues (such as the signals released by the niche). Dysfunctional FAP regulation by alterations of these regulatory conditions may severely deteriorate muscle health, affecting both muscle function and metabolism.^11, 15, 45, 46^ As for the latter, FAPs may influence the metabolic activity of the muscle since ectopic adipocytes are associated with impaired insulin sensitivity, metabolic syndrome and type 2 diabetes (T2D).^47, 48, 49^ In this regard, it is intriguing to speculate as to what extent systemic circulating factors may alter FAP differentiation, including high glucose conditions shown to induce adipogenesis in muscle-derived stem cells.^50^ Moreover, deregulated FAP activity can contribute to the increased intramuscular adipose tissue (IMAT) observed in aged muscles^51^ and in patients with RA,^52^ COPD^53^ and cancer cachexia (Figure 5).^54^ Thus, FAPs may hold a dual role in which they provide important paracrine stimuli to support SC function in healthy muscle, while contributing to ectopic adipose accumulation in pathological conditions^48, 55, 56^ (Figures 2 and 5), thereby leading to reduced insulin sensitivity^48, 57^ and decreased muscle function.^58, 59^

### Role of SCs, FAPs and immune cells in neuromuscular disorders

Most neuromuscular disorders are initially alleviated by the regenerative potential of skeletal muscles. For instance, in Duchenne muscular dystrophy (DMD), compensatory regeneration at earlier stages of disease tends to counter the degeneration of dystrophin-deficient myofibers. While, at least in mouse models of muscular dystrophy, this reactive regeneration resembles muscle repair following injury, as the disease progresses the asynchronous waves of regeneration and the changes in niche caused by chronic regeneration eventually bias the repair toward pathogenic fibrosis and fat deposition.^60, 61^

Optimal regeneration entails a sequence of events that ensures temporally coordinated interactions between SCs, FAPs and cells of the immune system. An initial activation of resident immune cells and the inflammatory infiltrate,^62^ is typically followed by the sequential activation of FAPs and SCs.^63, 64^ Progressive impairment of the interplay between SCs, FAPs and immune system is emerging as a key event in switching regeneration from compensatory to pathogenic.^65, 66^ In particular, FAPs appear to play a central role in this switch.^59, 67^

**Figure 4:**
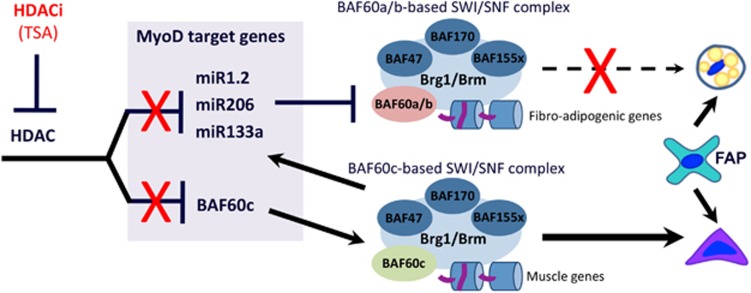
HDACs control an epigenetic network that determines FAP ability to support either regeneration or fibro-adipogenic degeneration. Inhibition of HDAC induced an upregulation of BAF60c that is engaged in the SWI/SNF complex leading to an increase of the myomiR expression and ultimately promoting a pro-myogenic phenotype in FAPs

Recent studies have revealed that FAPs from dystrophic muscles of mdx mice – the DMD mouse model – retain a phenotypic and functional bipotency, as they can either support compensatory regeneration at early stages of disease progression, or mediate fibrotic and fat deposition (Figure 4).^12, 16^ This alternative phenotype is regulated at the epigenetic level by a network formed by muscle-specific microRNA (the myomiRs miR-1.2, miR-133 and miR-206) that target key subunits of the SWI/SNF chromatin-remodeling complex. In particular, expression of myomiRs correlates with the ability of FAPs to support SC-mediated myogenesis and to adopt a myogenic phenotype at the expense of the fibro-adipogenic lineage.^16^ This outcome is typical of compensatory regeneration at early stages of disease progression and likely reflects the action of cues from a regeneration-conducive SC niche that resolve FAP bipotency into the pro-myogenic phenotype. Saccone *et al.*^16^ showed that myomiRs selectively target two variants of the BAF60 subunit of SWI/SNF complex (BAF60A and B), which activate chromatin remodeling at fibrotic and adipogenic loci. This leads to the selection of the alternative variant – BAF60C – which promotes chromatin remodeling at muscle loci^68^ and mediates FAP commitment to the myogenic lineage^16^ (Figure 4).

Importantly, this intracellular network is controlled by histone deacetylases (HDACs),^69^ whose activity is constitutively active in DMD muscles.^70^ In normal conditions (i.e., physiological regeneration) reversible HDAC-mediated repression of myomiR allows the expression of BAF60A and B variants and supports maintenance of bipotency in FAPs. In dystrophic muscles, constitutive HDAC activity represses BAF60C and myomiR and favors the expression of BAF60A and B, which direct the fibro-adipogenic phenotype of FAPs. Interestingly, this dynamic HDAC-mediated regulation of phenotypic and functional bipotency of FAPs is observed at early, but not late stages of disease progression in mdx mice, and accounts for the restriction of the beneficial effects of HDAC inhibitors at early but not late stages of disease.^12^

Future studies should establish how FAP bipotency, and relative resolution, is regulated by cues from the regenerative microenvironment and should elucidate the signals that controls reciprocal interactions between FAPs, immune system and SCs within physiological and pathological contexts. For instance, it would be interesting to evaluate the contribution of FAPs to the excessive levels of transforming growth factor-*β*2 (TGF*β*2), which are induced by elevated canonical Wnt signaling in dystrophic muscles and affect the behavior of SCs.^71^

As the human counterpart of FAPs has been identified as PDGFR*α*^+^ cells in healthy and diseased muscles,^14^ the functional and molecular characterization of human FAPs can provide novel interesting targets for the development of pharmacological treatment of muscular diseases.

### Role of SCs, FAPs and immune cells during ageing and metabolic dysfunctions

Aging is associated with an accelerated loss of skeletal muscle mass (sarcopenia) and with a reduced regenerative capacity of the musculature, leading to a loss of strength and function.^72, 73, 74^ In rodents, the aging muscle and SC niche has been shown to disrupt SC function and myofiber regenerative capacity.^75, 76, 77, 78^ Local changes in the SC milieu include a number of events that can alter signaling in SCs. For instance, constitutively elevated activation of p38 kinase, STAT3 activity and reduced Notch signaling has been observed in aged SCs.^46, 47, 79, 80, 81, 82^ Likewise, increased TGF*β* activity and induction of the cyclin-dependent kinase inhibitors associated to inhibition of cellular proliferation, such as p15, p16 and p21, have been reported as potential triggers of SC senescence.^79, 80, 81^

The events described above likely depend on extensive changes in the SC niche, including deregulated activity and number of FAPs or additional cellular components, such as fibroblasts and adipocytes,^74^ that originate from FAP differentiation. In a mouse model of young and old mice sharing the circulatory system (heterochronic parabiosis model) aged SCs were rejuvenated by exposure to a young systemic environment suggesting that the tissue-specific stem cells retain their proliferative potential, but that the aged systemic environment prevents full activation.^76^ These findings have been sparsely investigated by *in vitro* studies on human primary cells, leading to contradictory results.^80, 82^ Age-induced changes in the systemic milieu include reduced local capillary network and endothelial cell apoptosis/senescence, which can lead to reduced secretion of SC stimulatory factors, impaired chemotaxis of immune cells and collectively a more negative balance between positive and negative regulators of SC activity. Recent evidence points to the importance of systemic concentrations of the circulating proteins such as oxytocin^83^ or growth differentiation factor 11 (GDF11),^84^ although it is currently controversial whether GDF11 levels decrease or increase with aging, as well as the relative efficacy of GDF11 supplementation in countering the functional decline of aged muscle and SCs.^85^

Interestingly, sarcopenia in rodents is not further accelerated during conditional ablation of Pax7^+^ SCs.^25^ However, despite the lack of direct effects on muscle fiber size, ablation of Pax7^+^ cells during sarcopenia generated increased levels of collagen deposition, preferentially in fast muscles,^25^ which could derive from fibrogenic differentiation of FAPs. In human skeletal muscle *in vivo* the SC content in type II muscle fibers is selectively reduced with aging, whereas the number of SCs in type I fibers remains similar to young individuals, following the pattern of a selective atrophy of type II muscle fibers.^86, 87^ Thus, while SC content does decrease during sarcopenia in both rodent and human skeletal muscle, it is not yet entirely defined to what extent the decrease in SC content can account for muscle atrophy or vice versa. Although this selective deterioration of type II fibers and their SC content in human skeletal muscle is partly reversible by resistance training,^87, 89^ the responsiveness of SCs to a single bout of resistance exercise is reduced with aging.^21, 88^ Even lifelong (endurance) exercise does not seem to prevent the decrement in type II fiber size or SC content compared to type I fibers.^90^ However, the amount of adipose infiltration in the old untrained muscle was larger than in the trained groups (unpublished observation, URM). It is therefore intriguing to speculate that changes in the muscle microenvironment or systemic environment related to inactivity or ageing can condition FAP phenotype and ability to release important paracrine cues to SCs and myofibers to support regeneration and muscle growth.

In addition to muscle atrophy, inactivity and ageing are commonly associated with increased adiposity, together leading to metabolic dysfunctions such as dyslipidemia, decreased insulin sensitivity, hyperglycemia and an increased risk of developing diabetes mellitus (i.e., T2D). Since skeletal muscle is the most abundant tissue of the body for glucose disposal, muscle sensitivity to insulin action is essential in development of whole body insulin resistance and hyperglycemia.^91^ Moreover, patients with T2D show a greater decline in muscle mass, muscle strength and functional capacity with aging.^92^ A common observation in conditions associated with impaired skeletal muscle insulin sensitivity is accumulation of ectopic lipids within (intracellular) and between (extracellular) skeletal muscle fibers^48, 56, 57, 93, 94^ (as illustrated in Figure 1), which is linked to reduced insulin sensitivity^48, 57, 94^ and decreased muscle function.^58^ Paradoxically, endurance athletes also display an elevated level of intracellular lipid (termed athletes-paradox), presumably serving as energy source during physical activity,^95^ although they also exhibit increased insulin sensitivity, as compared to healthy untrained subjects.^96, 97^ In contrast, the IMAT (i.e., adipose tissue within a muscle but located outside the myofiber) is to our knowledge not increased in athletes and is associated with reduced insulin sensitivity in both healthy^47^ and obese^48^ subjects as well as in acromegaly patients.^49^ Although the origin of IMAT is not yet known,^56^ murine muscle-derived stem cells have been shown to undergo adipogenic differentiation upon exposure to elevated glucose levels *in vitro*,^50^ providing a potential link between adipocyte accumulation and the systemic milieu. Moreover, while previous work proposed that IMAT can originate from trans-differentiation of myogenic stem cells,^50, 56, 98^ increasing evidence suggests that FAPs could constitute the mesenchymal stem cells responsible for IMAT accumulation in skeletal muscle.^12, 14, 99, 100^ In addition, FAP-derived adipocytes may have reduced insulin sensitivity compared to conventional adipocytes, suggesting that accumulation of FAP-derived adipocytes may contribute to a compromised peripheral insulin sensitivity.^101^

Presently, the role of FAPs and their interplay with SCs, eosinophils or macrophages in relation to development of T2D is unknown. However, dysfunctional regulation of FAPs mediated by changes in IL-4 signaling may influence skeletal muscle homeostasis. Interestingly, IL-4 levels have been reported to be positively associated with insulin sensitivity^102^ and IL-4 promoter polymorphisms have been associated with T2D.^103^ Moreover, IL-4-mediated signaling may prevent adipogenesis in muscle and adipose tissue.^102, 104^ In white adipose tissue, IL-4 released from tissue-resident eosinophils regulates the presence of M2 macrophages,^31^ which are positively associated with insulin sensitivity.^105^ When genetically depleting IL-4 secreting eosinophils the content of the M2 macrophage phenotype in adipose tissue is reduced and this is associated with a substantial decrease in glucose tolerance and insulin sensitivity.^31^ These changes are less explored in humans, however, increased expression of the inflammatory macrophage phenotype marker; CD11c in skeletal muscle of T2D patients has been reported.^106^

Reduced insulin sensitivity is often associated with aging^107^ and in chronic diseases that cause cachexia.^108, 109^ Patients suffering from cachexia or patients affected by metabolic syndrome may display elevated levels of glucocorticoids^110^ that may influence IL-4 secretion. Interestingly, elevated glucocorticoid levels (such as those reached upon dexamethasone treatment) in mice increase the adipogenic differentiation of FAPs, which is otherwise suppressed through IL-4-mediated signaling.^15^ The effect of dexamethasone treatment on FAP adipogenesis may relate to suppression of eosinophil release of IL-4^111^ as suggested by Dong *et al.*^15^ Thus, elevated levels of glucocorticoids during disease (either as medical treatment or endocrine release) could increase adipocyte accumulation in skeletal muscle through adipogenic differentiation of FAPs and hereby negatively impact the insulin sensitivity of skeletal muscle.^47, 48^ Given that steroids are used in the treatment of many muscular disorders (including DMD), the interactions between glucocorticoids, FAP, SCs and cells of the immune system deserve future investigation.

### Role of SCs, FAPs and immune cells in cachexia

Cachexia consists of an accelerated muscle loss that is associated with chronic diseases, complicates their recovery and is an independent predictor of morbidity and mortality.^112, 113, 114^ Skeletal muscle wasting is a common phenomenon in cancer patients,^115, 116, 117, 118, 119^ and cancer-related muscle loss affects up to 80% of patients with advanced cancer, leading to poorer prognosis, reduced treatment response and increased risk of complications during surgery and chemotherapy. Ultimately, cachexia accounts for >20% of all cancer-related deaths.^120, 121, 122, 123, 124^

Since both SCs and FAPs may influence muscle homeostasis and growth, their interactions can be implicated in cachexia. In rodents, cancer cachexia is associated with muscle damage and deregulation of Pax7 expression in SCs and interstitial cells, through increased NF-*κ*B activity,^125^ suggesting that NF-*κ*B may contribute to muscle wasting in cancer.^125^ In muscle biopsies from pancreatic cancer patients with accelerated weight loss, an increased number of Pax7^+^ cells was observed, indicating that cancer cachexia is associated with an expansion of the myogenic precursor pool.^125^ Expansion of the Sca1^+^ cell population that resemble mouse FAPs, was also observed in cachectic muscles from tumor-bearing mice. Interestingly, this Sca1^+^ population preferentially adopt the myogenic lineage under the influence of tumor environment, by expressing the SC-specific transcription factor Pax7, which is induced by serum factors from cachectic mice and patients, in an NF-*κ*B-dependent manner; however, completion of differentiation was also inhibited by the persistent expression of Pax7.^125^ Restoring the myogenic potential of these cells by Pax7 downregulation or by ectopic expression of MyoD, promoted cell differentiation and fiber fusion and reversed muscle wasting.^125^ These results reiterate the concept that Sca1^+^ cells have a latent myogenic potential that can be induced by environmental signals, for example, the elevated levels of cytokines from systemic inflammation. Since, Pax7 expression is regulated by inflammation-induced signals,^126^ these data reveal again the important interactions between the immune system and FAPs in the control of muscle regeneration. However, it is important to note that Sca1^+^ cells might only account for a subpopulation of FAPs, or even a distinct population of cells induced in tumor-bearing conditions. A recent study not only identified stromal cells by expression of fibroblast activation protein *α* (but also uniformly expressing FAP markers such as CD90, PDGFR*α* and Sca1), and observed a depletion of these stromal cells to be underlying the cancer-induced cachexia.^127^ Specifically, these stromal cells appeared to maintain muscle size through paracrine secretion of follistatin, which in turn reduced the muscle expression of ubiquitin ligases such as atrogin-1 and muscle RING-finger 1 (MuRF1) involved in muscle protein breakdown.^127^ It remains to be investigated if factors released directly by the tumor, alterations in immune cell content/function or other mechanisms may underlie the depletion of the stromal cells in skeletal muscle during cancer cachexia. If these stromal cells are indeed FAPs, an alternative explanation for the cell content depletion during cachexia may relate to an increased adipogenic differentiation of the FAPs. In this regard the progression of cachexia has been associated with an increased amount of intramuscular lipid droplets,^54^ and although the source of these is not identified they could originate from FAPs.

Multiple chronic diseases are also associated with elevated systemic inflammation including RA.^128, 129, 130, 131, 132, 133^ In RA patients, loss of muscle strength is associated with RA duration rather than with chronological age, in contrast to the decline with age observed in the general population.^134^ This indicates a disease-related effect on muscle strength that surpasses the effect of aging. Increased IMAT, potentially originating from FAPs, is observed in the muscle of RA patients and this reduction in muscle density associates with greater joint destruction.^52^ Furthermore, type II fiber atrophy in RA patients has been reported,^135^ but compared to patients with osteoarthritis the absolute numbers of SC^136^ and their *in vitro* regenerative potential^137^ were not different in RA patients.

COPD is another frequent cause of disease-related cachexia and premature death worldwide.^138^ COPD is often accompanied by pronounced muscle wasting (atrophy of both type I and II fibers has been reported^139^) and metabolic dysfunction.^138, 140^ Notably, the loss of muscle mass is an independent predictor of mortality in COPD patients.^1, 112^ The alterations in immune cell function and the chronic inflammatory condition is believed to be a substantial contributor to the loss of muscle mass along with an impaired regenerative capacity and cellular apoptosis.^138, 140^ SC content is not altered in the muscles of COPD patients compared to controls; however, primary SCs isolated from COPD patients displayed a delayed activation in culture and decreased expression of myosin heavy chain expression during myotube formation compared to controls.^141^ Interestingly, the increased number of central nuclei observed in the muscles of COPD patients with preserved muscle mass, as compared to those who lost muscle mass,^141, 142^ suggests that the ability of the muscle to regenerate could attenuate the extent of COPD-related cachexia.

**Figure 5:**
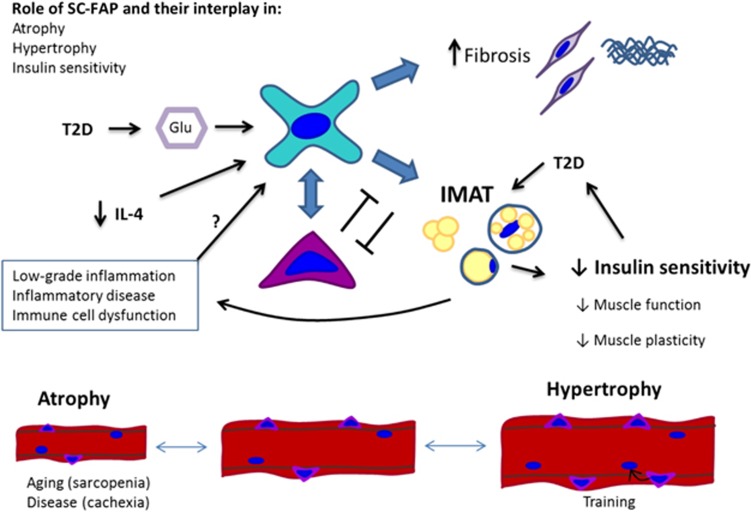
Schematic illustration showing potential role of FAP-SC and their interplay in muscle: atrophy (as observed with aging and disease), hypertrophy and insulin sensitivity. IL-4, interleukin-4; IMAT, intramuscular adipose tissue; T2D, type 2 diabetes

Collectively, the potential role for SC and FAPs in relation to muscle loss due to chronic inflammatory diseases is a matter of current and future investigations (Figure 5).

## Exercise as a Strategy to Improve Muscle Health – Stimulation of SC and FAPs by Exercise

Skeletal muscle is a highly plastic tissue that adapts to stimuli, by proportionally adjusting mass and strength (resistance training) or aerobic capacity (endurance training) in response to exercise. Both acute and prolonged resistance exercise stimulates the proliferation of SCs in healthy subjects,^143, 144, 145, 146, 147^ and even non-hypertrophying endurance exercise can induce proliferation of at least some SC populations.^7^ Although the potential impact of different training modalities on the immune-FAP-SC network are described separately below, it is important to note some remarkable differences, and the relative impact on the network, between physical exercise and the pathological conditions described in the previous paragraphs. For instance, in most pathological conditions FAPs are activated by physical injury, which triggers extensive changes in the microenvironment, (e.g., myofiber degeneration in muscular dystrophies) or by elevated systemic concentration of inflammatory cytokines (e.g., cachexia); by contrast, during training most of these signals are absent – except for the case of strenuous exercise – and the predominant changes occurring in exercised muscles are of metabolic (redox alterations) or biomechanical (contraction/relaxation cycles) nature. As FAPs occupy an interstitial position they are a great candidate as cell types that sense these changes and transmit them to SCs via specific cues. In this regard, recent work reported that SIRT1, a NAD(+)-dependent HDAC known as redox and nutrient sensor, promotes the metabolic switch from fatty acid oxidation to glycolysis during the SC transition from quiescence to proliferation.^148^ Moreover, SIRT1 regulates autophagic flux in SCs to cope with the high bioenergetic demands during the activation process.^149^ Finally, SIRT1 connects changes in SC metabolism with changes in the transcriptional machinery toward myogenic commitment of the SC.^148^ This reprogramming of cellular metabolism decreases intracellular NAD(+) levels and the activity of the HDAC SIRT1, leading to elevated H4K16 acetylation and activation of muscle gene transcription. Future studies should establish whether SIRT1 is activated by FAP-derived signals.

## Resistance and Endurance Training, Muscle Hypertrophy and Insulin Sensitivity

Although the role of SCs during myofiber regeneration has been extensively studied and reviewed,^8, 22, 23, 150, 151, 152, 153^ it is debated whether SCs possess a role in myofiber hypertrophy in the adult muscle.^154, 155^ In earlier studies, irradiation was used to ablate SC activity, whereby overload induced hypertrophy in rodents was prevented,^154, 156, 157^ indicating a direct linkage between SCs and myofiber hypertrophy. However, a later study opposes this contention by showing an intact hypertrophic response in myofibers of SC-depleted muscles in rodents.^26^ Although these findings from conditional knockout mice seem to reject the hypothesis that SCs are essential for hypertrophy, more recent results indicate that the lack of SCs can attenuate myofiber hypertrophy in the later phases of an overload period.^19^ The latter is supported by other studies suggesting an important role for SC in myofiber growth and myonuclei accretion.^24, 158, 159^ In agreement, IGF-1, which can accentuate resistance training-induced muscle hypertrophy,^160^ may in part act through increased proliferation and differentiation of SC to support myofiber growth.^161^ This is supported by the increased expression of IGF-1 splice variants in human SCs following eccentric resistance exercise.^162^

In several human studies, robust increases in the number of SCs has been shown both acutely^21, 163, 164^ and following prolonged ^143, 144, 165, 166, 167, 168^ resistance exercise in both young and old humans.^87, 88, 89^ In contrast, SC proliferation following resistance exercise may be impaired during ageing and in patients affected by chronic muscular disorders, with current evidence suggesting that this impairment originates from alterations in cues from the SC niche or the systemic environment.^20, 21, 76, 83, 88^ However, long-term resistance training can reverse the SC distribution in elderly muscle toward that of young muscle.^169^ Knowledge about the regulation of FAPs in relation to resistance training and hypertrophy is lacking, but in rodents the involvement of a SC-FAP interplay in successful muscle regeneration after muscle damage has been convincingly demonstrated.^9, 11, 28^ Although damage/regeneration is not a prerequisite for resistance training adaptations,^170^ the rodent findings combined with human SC data suggest that SC-FAP interplay may have a central role in resistance training adaptations (Figure 5). Furthermore, detraining in elderly is accompanied by an increased amount of muscle fat infiltration which can be reversed by resistance training,^171^ and reducing ectopic fat accumulation may enhance myofiber anabolic signaling.^46^ Collectively, this area awaits further investigation in humans; however, the present body of data indicates that FAPs may be regulated with resistance exercise-induced hypertrophy. One mechanism may relate to the recently identified circulating hormone Meteorin like, which is secreted from skeletal muscle upon exercise and triggers IL-4, and IL-13 production by eosinophils in adipose tissue. These cytokines cause alternative activation of M2 macrophages^29, 172^ as shown in Figure 2, and are also involved in the regulation of FAP activity, further providing a tentative link between exercise, immune cell activation and FAP regulation.

Endurance exercise increases insulin sensitivity and glucose tolerance,^173, 174, 175, 176^ for example, via increased protein expression of insulin receptor substrate-1 (IRS-1) and GLUT4^176, 177^ in skeletal muscle. Although increased energy expenditure through endurance training reduces accumulation of adipose tissue, insulin sensitivity and glucose tolerance are improved independent of weight loss.^178^ The SC response to endurance training has only been evaluated in a few human studies, and increased SC numbers are reported in most,^179, 180, 181^ but not all^182^ of these, mostly in the absence of muscle fiber hypertrophy. Recently, hypertrophy of both type I and IIa fibers was observed following 12 weeks of aerobic training, with a concomitant increase in SC number only in type I fibers.^181^ Furthermore, non-hypertrophying endurance exercise can induce proliferation of SC populations in hybrid fibers (type I/II) without effect on the SC content of type I or II fibers.^7^ Generally, these data indicate a role for SC proliferation and turnover in muscle maintenance even in the absence of fiber hypertrophy, which is in line with the suggested role for SC-FAP interplay in regulation of a healthy muscle.

## Perspectives and Conclusions

FAPs are emerging as a 'cellular filter' between external perturbations (either local or systemic changes in physical, metabolic and inflammatory cues) and the effectors of the muscle regeneration machinery – the SC. Depending on the nature of the perturbations FAPs appear to adopt specific phenotypic and functional properties indicating a highly heterogeneous cell population.^183^ Thus, FAP heterogeneity and the dynamic transition from a physiological to compensatory or pathological subpopulation appears as a key issue to investigate in future studies. In particular, the anatomical derivation of FAPs might reveal important differences. For instance, in the presence of physical insults, the ensuing vessel injury or transient ischemia might direct a composition of FAPs that is phenotypically and functionally different from cells derived by the expansion of resident interstitial FAPs.

Overall, the elucidation of the interplay between SCs, FAPs, their niche and immune cells might have an impact not only in the discovery of interventions toward restoring muscle function and correcting metabolic dysregulation in pathological conditions, but also to improve muscle anabolism and insulin sensitivity, which commonly decreases during aging, inactivity and certain disease states.

## Abbreviations

FAP: fibro-adipogenic progenitor
SC: satellite cell
Sca1: stem cell antigen 1
PDGFR*α*: platelet-derived growth factor receptor alpha
FACS: fluorescence-activated cell sorting
IGF-1: insulin-like growth factor 1
HGF: hepatocyte growth factor
NO: nitric oxide
STAT: signal transducer and activator of transcription
NAD: nicotinamide adenine dinucleotide
IL-4: interleukin-4
SIRT1: sirtuin 1
COPD: chronic obstructive pulmonary disease
RA: rheumatoid arthritis
DMD: Duchenne muscular dystrophy
SWI/SNF: SWItch/sucrose nonfermentable
HDAC: histone deacetylase
TGF*β*: transforming growth factor beta
GDF: growth differentiation factor
Pax7: paired box transcription factor 7
MyoD: myogenic differentiation
Wnt: wingless type
MuRF1: muscle RING-finger protein 1
T2D: type 2 diabetes
IMAT: intramuscular adipose tissue
NF-*κ*B: nuclear factor kappa-light-chain enhancer of activated B cells
